# Evaluation of the effect of cervical spine bone distribution on fixation in ankylosing spondylitis

**DOI:** 10.3389/fbioe.2025.1430047

**Published:** 2025-02-27

**Authors:** Beiyang Wang, Jian Tang, Zhiqiang Wang, Chen Deng, Junqiao Lv, Fang Gao, Xiaoyan Xiong, Lin Sun

**Affiliations:** ^1^ Third Hospital of Shanxi Medical University, Shanxi Bethune Hospital, Shanxi Academy of Medical Sciences, Tongji Shanxi Hospital, Taiyuan, China; ^2^ College of Mechanical and Vehicle Engineering, Taiyuan University of Technology, Taiyuan, China; ^3^ Key Laboratory of Advanced Transducers and Intelligent Control System, Ministry of Education, Taiyuan University of Technology, Taiyuan, China

**Keywords:** finite element, ankylosing spondylitis, spine, fracture, osteoporosis

## Abstract

**Purpose:**

The distribution of cervical bones in ankylosing spondylitis (AS) differs from that of the normal cervical spine. Traditional simulation methods often yield inaccurate results in finite element analysis. The current study aimed to construct ankylosing spondylitis cervical spine fracture (ASCF) models based on Hounsfield Unit (HU) values to analyze the effects of different fixation approaches.

**Methods:**

Quantitative HU measurements of cervical vertebrae and lateral masses were obtained from CT scans of 20 patients with AS. A finite element model of ASCF was constructed based on HU values and was compared with a traditional ASCF model from multiple perspectives. Additionally, three ASCF models were used to compare the effects of various fixation approaches. A meta-analysis of screw loosening rates was conducted to further validate the efficacy of the models.

**Results:**

The HU value of the cervical lateral mass in AS is higher than the corresponding mass in the vertebral body. Finite element analysis results indicated that the anterior approach is less stable compared to other approaches, as evidenced by the maximum stress (MS) value of the screw and the maximum displacement (MD) of the entire model. These findings were corroborated by the meta-analysis of screw loosening rates in ASCF.

**Conclusion:**

ASCF exhibits an uneven distribution of cervical bone, with more severe osteoporosis in the anterior cervical spine. Consequently, simple anterior approaches to fixation may lead to screw loosening in ASCF.

## 1 Introduction

Ankylosing spondylitis (AS) of the cervical spine is primarily characterized by osteoporosis and ligamentous ossification, which show significant differences from the bone distribution patterns observed in typical cervical vertebrae (Fitzgerald et al., 2020). The occurrence of ankylosing spondylitis of the cervical spine (ASCF) is often associated with a high incidence of complications and mortality (Luksanapruksa et al., 2019; Sun et al., 2023). There are three surgical fixation approaches for ASCF: anterior, posterior, and combined posterior-anterior (CPA) (Gao et al., 2021; Shen et al., 2022; Liu et al., 2023). Several studies have suggested that anterior approaches could effectively secure internal fixation, thereby mitigating the risk of spinal nerve damage (Chen et al., 2024). However, clinical observations have indicated an increased incidence of screw loosening following simple anterior approaches (Zhang et al., 2023a). Therefore, conducting a biomechanical analysis of the effects of different fixation approaches is necessary.

Presently, the majority of finite element models of ASCF are constructed employing CT imaging from healthy volunteers (Robinson et al., 2018; Liu Y. et al., 2021). In these models, cortical and cancellous bone materials are uniformly assigned, and ligament ossification is modeled using cortical bone material. However, research on the specific bone distribution in AS is scarce (Ward and Tan, 2023), and using these traditional simulation methods can lead to significant deviations in biomechanical outcomes.

HU values have been widely used clinically as an indicator of bone status (Schreiber et al., 2011; Wang et al., 2022; Marques et al., 2023). In the present research, HU values of the cervical vertebrae and lateral masses in 20 AS patients were determined and their patterns analyzed. Subsequently, bamboo-like models were developed from CT data of ASCF patients, material values were assigned based on HU values, and these models were used for biomechanical analysis. Additionally, a meta-analysis was conducted on screw loosening rates associated with these approaches to further validate the efficacy of the models. This study proposes an innovative method for simulating vertebral osteoporosis and ossified ligaments in ASCF and thus offers vital theoretical support for the biomechanical analysis and selection of different fixation approaches in the treatment of ASCF.

## 2 Methods

### 2.1 Measurement of HU values in the cervical spine of AS patients

**FIGURE 1:**
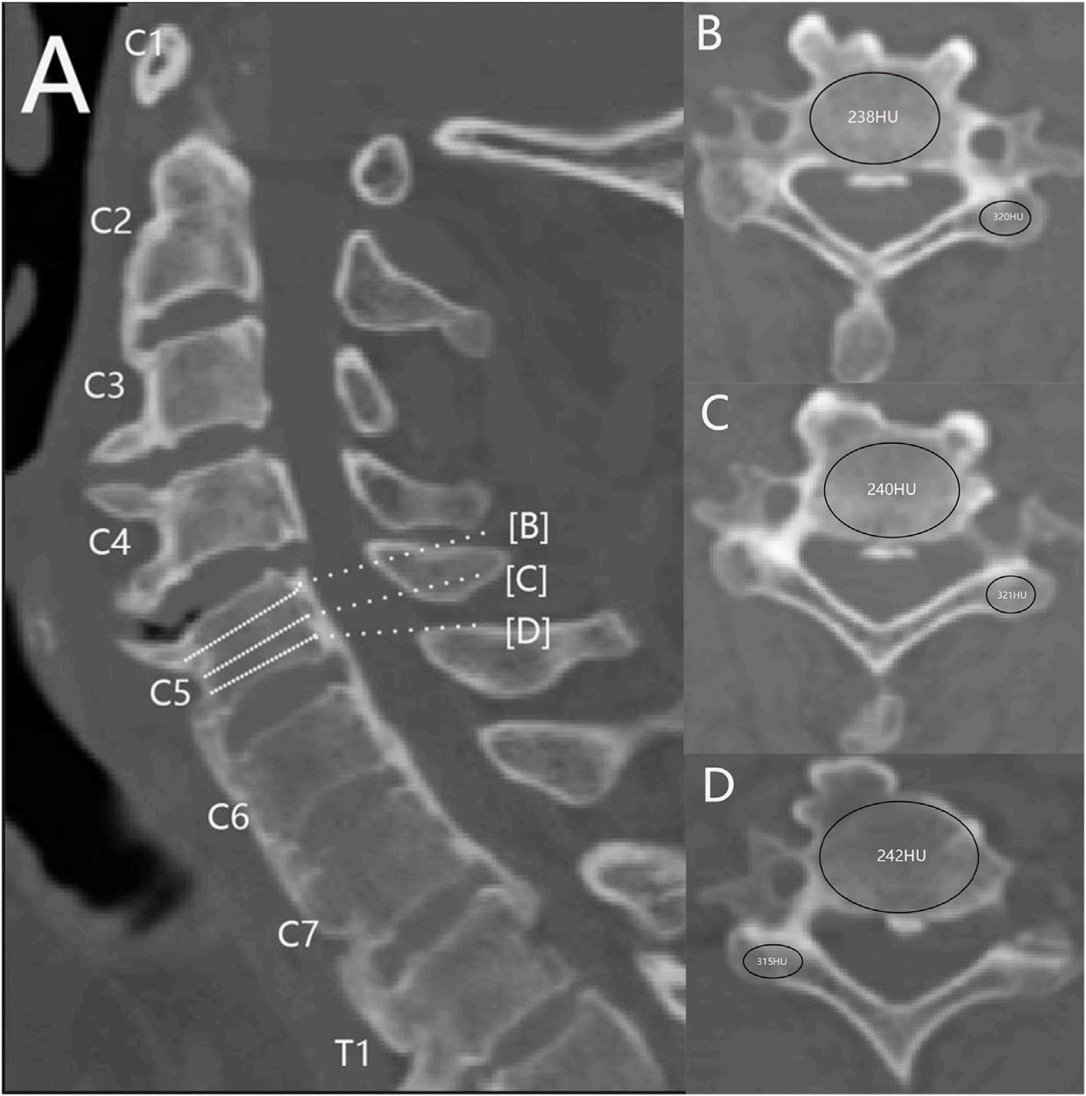
Measurement of the HU value of cervical spine AS. **(A)** Mid-sagittal position. **(B**–**D)** ROIs of vertebral bodies and lateral masses at different levels.

The Syngo imaging system was employed to determine the HU values. All patients included in the study were diagnosed with ASCF based on imaging data. Patients with spinal brucellosis, tumors, or tuberculosis were excluded from the study. Overall, 20 patients (15 male and 5 female subjects; average age, 47 ± 10 years) were enrolled to the current study. The HU values were determined within three regions of interest (ROIs) in the maximum elliptical layer of the vertebral body and lateral mass. These ROIs were selected at the middle of each site and the axial planes immediately above and below the cortical bone (Wang et al., 2023). The average HU value, derived from three ROIs, was considered representative of the vertebral body and lateral mass. The ROI was selected in order to include as much trabecular bone as possible and avoid cortical bone and heterogeneous areas (Figure 1). CT imaging data from three ASCF patients were utilized for model construction based on these HU values.

### 2.2 Finite element models

#### 2.2.1 Establishment of ASCF models based on HU values

**FIGURE 2:**
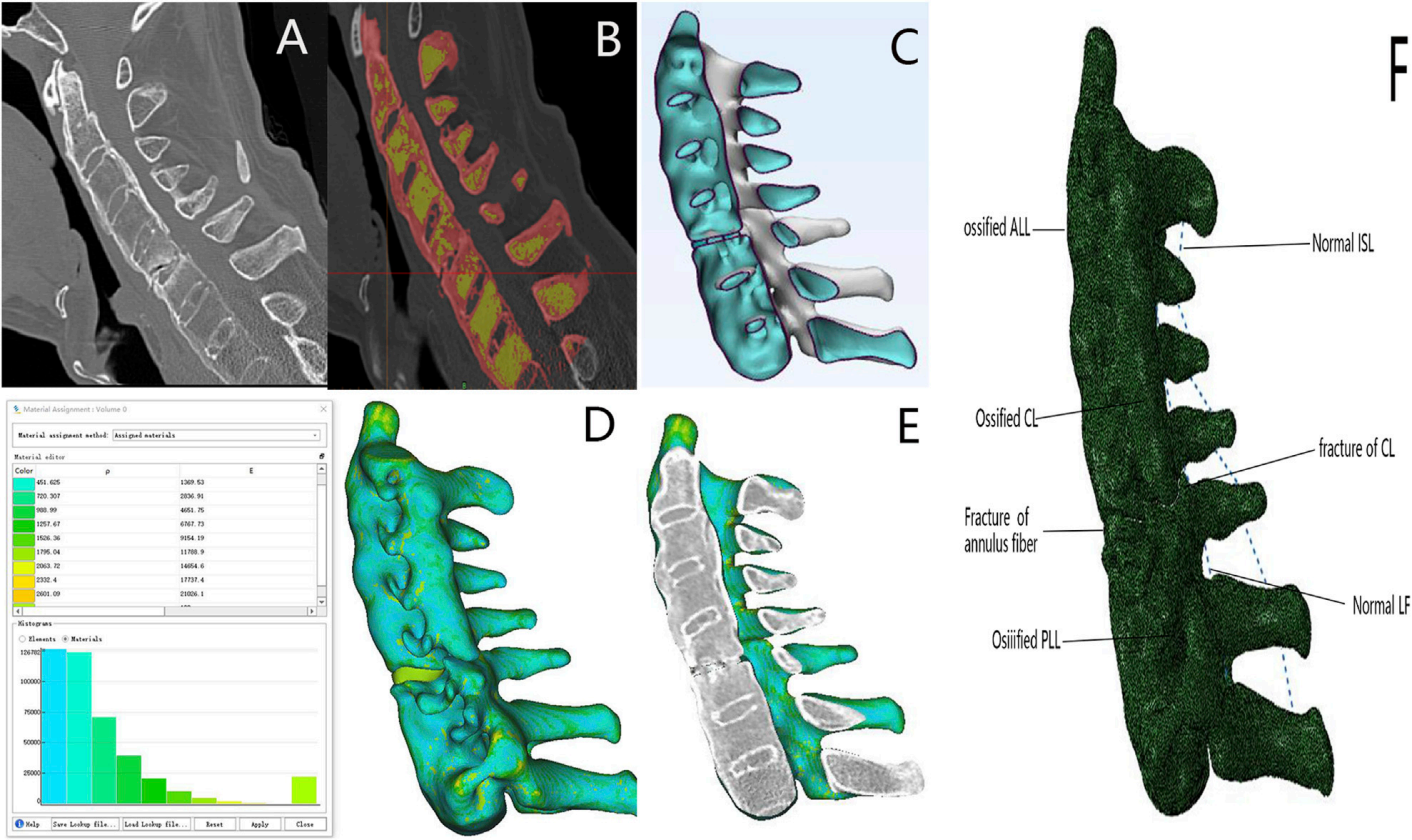
ASCF model established based on the HU value. **(A)** ASCF sagittal imaging. **(B)** Ossified ligaments and vertebral masks constructed using Mimics software. **(C)** Overall shape of the ASCF model. **(D)** Model shape based on the HU value assignment model. **(E)** ASCF model sagittal and CT sagittal images are consistent. **(F)** Model of the ASCF ligament.

This study was approved by the Ethical Committee of Shanxi Bethune Hospital, Taiyuan, and prior informed consent was obtained from each patient. Patients’ CT data were used to perform operations such as smoothing and filling of holes, and calculating volume based on the HU value range of bone CT. A multi-shell structure was constructed using Mimics version 23.0 based on CT data obtained from patients with ASCF. The outer shell comprised the outer layer of the vertebrae and the ossified anterior longitudinal ligament (ALL), posterior longitudinal ligament (PLL), capsular ligament (CL), and annulus fibrosus; the inner shell represented the nucleus pulposus structure (Figure 2). The mesh size of the model was set to 1 mm, and the HU value of each volume mesh was automatically recognized. The model material properties were assigned based on the HU value bone density elastic modulus formula using Mimics version 23.0, with the following relationships: ρ = 1.6 × HU, E = 0.09882 × ρ1.56, and ν = 0.3 (Rho et al., 1995; García-Vilana et al., 2023). Subsequently, spring elements in Abaqus were used to model the ligamentum flavum (LF) and interspinous ligament (ISL).

For developing fracture dislocation models, interactive translation and rotation were employed in 3-Matic to reduce the fracture models. Thereafter, Boolean operations were performed on the reduced model and the fixation. The model was simulated for validation studies and moved back to its original position using interactive translation and rotation. Finally, HU values were assigned to the dislocation model in Mimics version 23.0, and the material-assigned dislocation model overlapped with the reduced model in Abaqus using spatial geometric constraints (Supplementary Figure S1). This process allowed us to create a dislocated ASCF model based on HU values.

#### 2.2.2 Establishment of the ASCF model by the traditional method

A normal cervical spine model was constructed using CT data collected from normal volunteers in Mimics version 23.0 (Supplementary Figure S2). Subsequently, cortical bone, cancellous bone, articular cartilage, and intervertebral discs were generated in 3-Matic. Spring elements, including ALL, PLL, LF, ISL, and CL, were constructed in Abaqus with specified material properties (Xie et al., 2013; Wu et al., 2019a; Wu et al., 2019b; Wu et al., 2017) (Table 1). The model’s validity was ascertained by comparing its mechanical behavior in the four directions—flexion, extension, lateral bending, and rotation—with published data.

**TABLE 1:** Normal cervical spine material properties.

Component	E (MPa)	ν
Cortical bone (Liang et al., 2022)	12,000	0.3
Cancellous bone (El-Rich et al., 2009)	500	0.3
Nucleus (Ouyang et al., 2019)	1	0.49
Annulus ground substance (Ouyang et al., 2019)	3.4	0.4
LF	15	0.3
ISL	10	0.3
ALL	7.8	0.3
PLL	10	0.3
CL	8	0.3
Annulus fibrosus	4	0.4

E, elastic modulus; ν, Poisson’s ratio.

To simulate osteoporosis and ligament ossification in ASCF, the materials for cortical and cancellous bone were reassigned, and the materials for ALL, PLL, CL, and annulus fibrosus were designated as cortical bone (Liu Y. et al., 2021; Wang et al., 2023) (Table 2).

**TABLE 2:** Materials and properties of the ASCF model determined using the traditional method.

Component	E (MPa)	ν
Cortical bone (Robinson et al., 2018)	8,000	0.3
Cancellous bone (Robinson et al., 2018)	100	0.3
Internal fixation (Hussain et al., 2009)	110,000	0.3
Ossification tissue (Wang et al., 2023)	8,000	0.3
Cage (Hussain et al., 2009)	3,500	0.3

### 2.3 Comparison of ASCF models established by two methods

The model’s shape and sagittal plane were compared using CT image data of patients with ASCF. Differences in the ASCF models constructed by the two methods were evaluated under all four conditions: flexion, extension, bending, and rotation. The lower surface of the vertebral body in the models was fully constrained, and a downward load of 50 N was applied above C2 with a torque of 2 Nm.

### 2.4 Finite element analysis of ASCF with different fixation approaches

**FIGURE 3:**
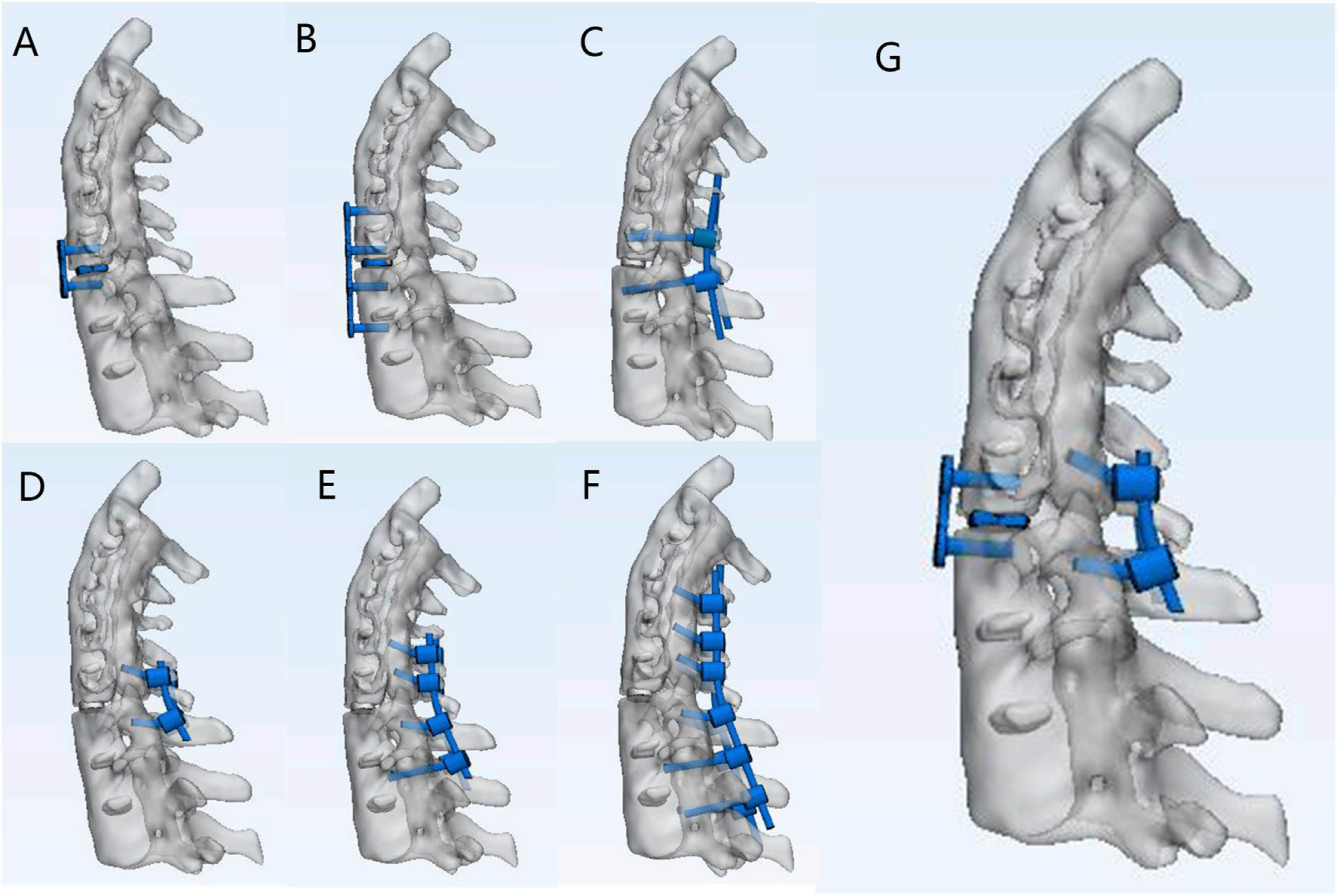
Different fixation approaches for C5–C6 fractures of ASCF. **(A)** A2. **(B)** A4. **(C)** P2* **(D)** P2. **(E)** P4. **(F)** P6. **(G)** A2P2.

Three ASCF models were developed based on HU values (Supplementary Figure S3) by simulating fractures at the C4-5, C5-C6, and C6-C7 segments. Each model was fixed using seven different approaches (Figure 3). The current study describes various models of spinal fixation, including two-segment anterior fixation (A2), two-segment posterior lateral mass screw fixation (P2), two-segment posterior pedicle screw fixation (P2*), and four-segment posterior lateral mass screw fixation (P4). Additionally, it covers anterior four-segment fixation (A4), posterior six-segment fixation (P6), and a combined approach of anterior two-segment fixation with posterior two-segment fixation (A2P2).

In this study, screws were simplified into cylindrical structures, with the interactions between the screws and the cervical vertebrae, as well as between the rods and the screws, set as binding. The P2, P4, and P6 models adopted the Magerl method: the nail insertion point was located 2–3 mm inside the midpoint of the lateral mass, and the nail insertion trajectory was parallel to the upper facet joint surface in the sagittal plane, with an outward inclination of 25°. The pedicle screws were inserted at an angle of 30°–45° to the vertical axis, ensuring that the screw plane was parallel to the upper and lower endplates. Additionally, pedicle screw fixation was utilized in all posterior fixations involving segments of the lumbar spine and below. In the posterior approach, bone blocks were used to replace the fractured and defective part of the anterior cervical spine. The lower surface of the vertebral body in the model was fully constrained, and a downward load of 50 N was applied above C2 with a torque of 2 Nm.

### 2.5 Further verification of the finite element analysis

Relevant studies were retrieved from several databases such as PubMed, Cochrane Library, Embase, and Web of Science. Due to the focus on the choice of cervical surgical fixation methods for AS, the search terms included (“Surgical Procedures, Operative” [Mesh] or Entry Terms), (“Spondylitis, Ankylosing” [Mesh] or Entry Terms), and (“Cervical Spine” [Mesh] or Entry Terms). A meta-analysis was conducted on the loosening rates of screws in different ASCF approaches to further validate the finite element results.

### 2.6 Statistical analysis

Statistical analyses were performed using statistical software SPSS version 23.0. Normality was assessed using the Shapiro-Wilk test. Data conforming to a normal distribution were analyzed using one-way ANOVA followed by Dunnett’s *post hoc* test. Non-parametric alternatives were applied for data that could not comply with normality assumptions. Statistical significance was set at *P* < 0.05.

## 3 Results

### 3.1 Distribution of HU values in the AS cervical spine

**FIGURE 4:**
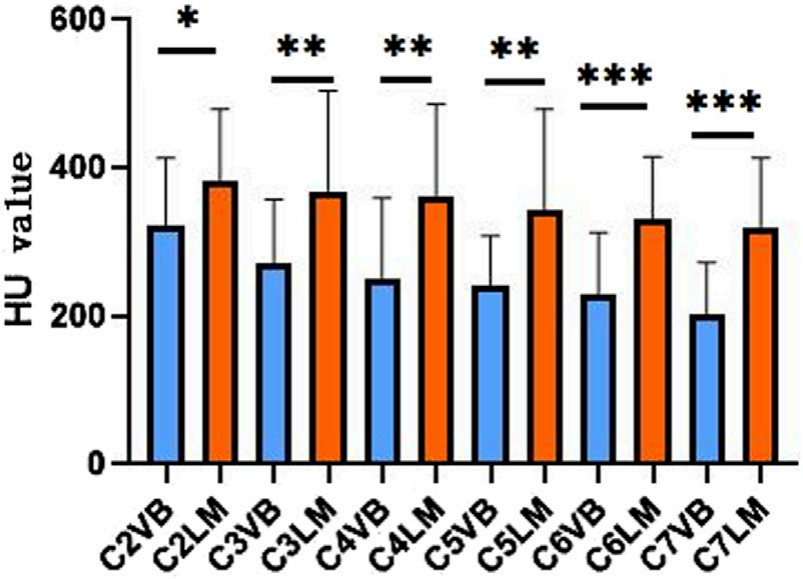
Comparison of the average HU value of vertebral and lateral masses at different segments of AS. VB, vertebral body; LM, lateral mass.

The HU values for the C2-C7 vertebral bodies and lateral masses are as follows: 322.91 ± 88.27, 382.85 ± 93.89, 270.6 ± 84.23, 368.9 ± 131.7, 250.45 ± 106.05, 361.16 ± 121.78, 240.05 ± 66.58, 342.25 ± 133.33, 228.18 ± 82.63, 330.86 ± 81.72, 201.95 ± 69.23, and 319.06 ± 92.02. The HU values of the AS cervical spine decrease sequentially from the superior to inferior levels, and the HU values of the same segmental vertebral body are smaller than those of the lateral mass (Figure 4). This pattern suggests a potential biomechanical gradient along the cervical spine.

### 3.2 Comparison of ASCF models constructed by two methods

The healthy volunteer cervical spine model aligns with the results of studies conducted by Panjabi et al. (2001), Wheeldon et al. (2006), and Ito et al. (2004)) in terms of flexion, extension, lateral bending, and rotational motion (Supplementary Figure S5). Upon calibrating the material properties to account for ossified ligaments and osteoporosis within the conventional model, the fractured upper and lower segments were thoroughly compared in the models developed using both methods. The discrepancies in the range of motion in all directions were minimal (<1°) (Supplementary Figure S6). However, the HU-based model more accurately resembles the actual ASCF, and its sagittal section can completely overlap with the sagittal section of the CT image (Figure 2E).

### 3.3 Comparison and meta-analysis validation of fixation effects of different ASCF approaches

The relationships between different fixation approaches in terms of maximum stress values for flexion, extension, bending, and rotation are as follows: A2 > A4, P2 > P4 > P6, A2 > P2*, A4 > A2P2, A2P2 > P4, P2 > P2*. Notably, the maximum stress values of internal fixation were localized in the fracture segment, with the highest stress values occurring at the contact area between the screw and the bone. Specifically, the maximum stress value of the screw in A2 during rotation was 62.9 MPa; it was significantly greater (46.66 MPa) than that in P2. The maximum stress value for P2* was 31.11 MPa, while for A2P2 it was 37.27 MPa. As the posterior fixation segment increased, the maximum stress value for P6 decreased to 27.15 MPa.

**FIGURE 5:**
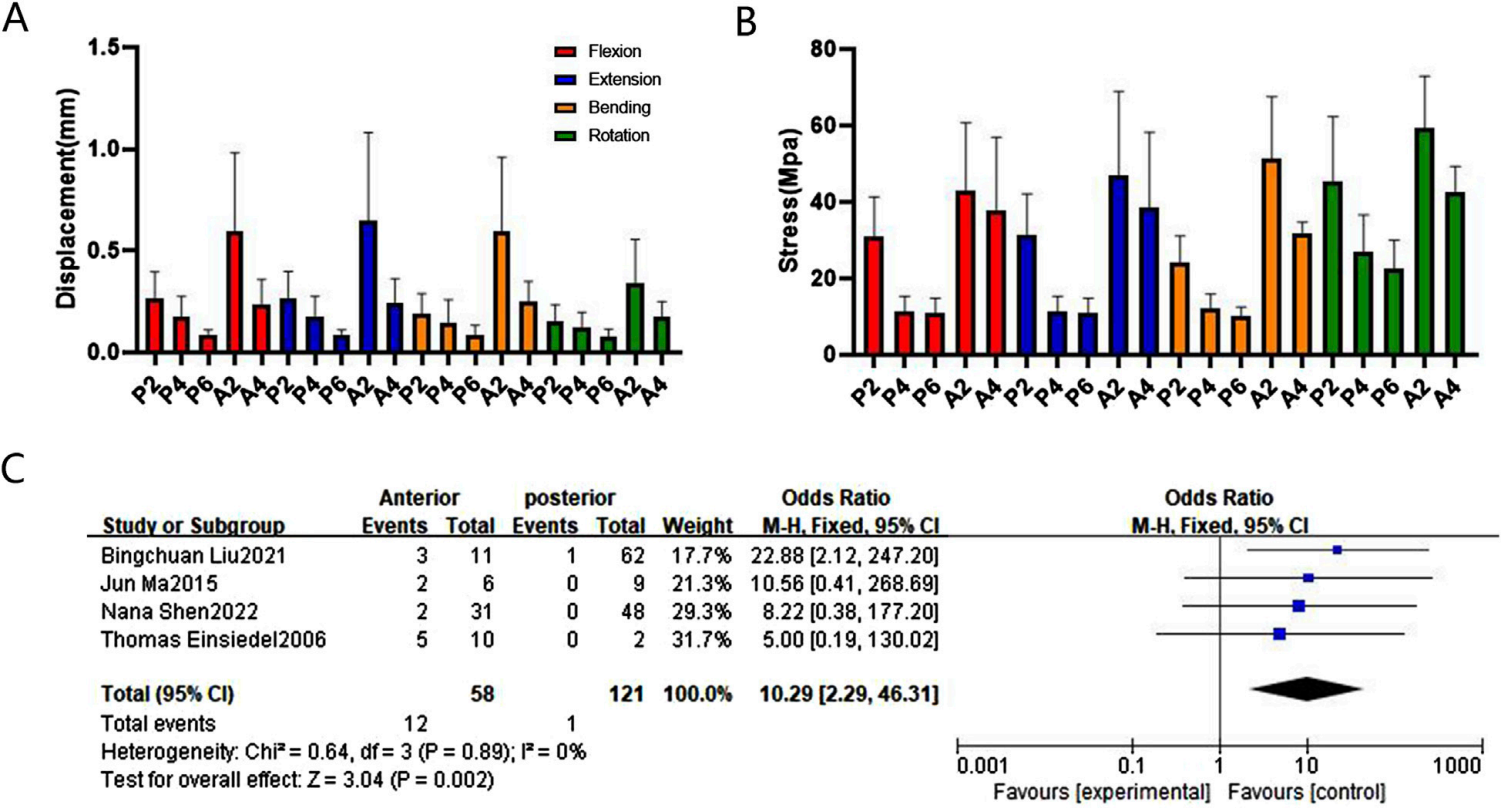
Comparison and verification of effects of anterior and posterior approaches. **(A)** Comparison of whole model displacement. **(B)** Comparison of screw stress values. **(C)** Meta-analysis of the screw loosening rate. Note: Blue points represent the effect size estimates.

**FIGURE 6:**
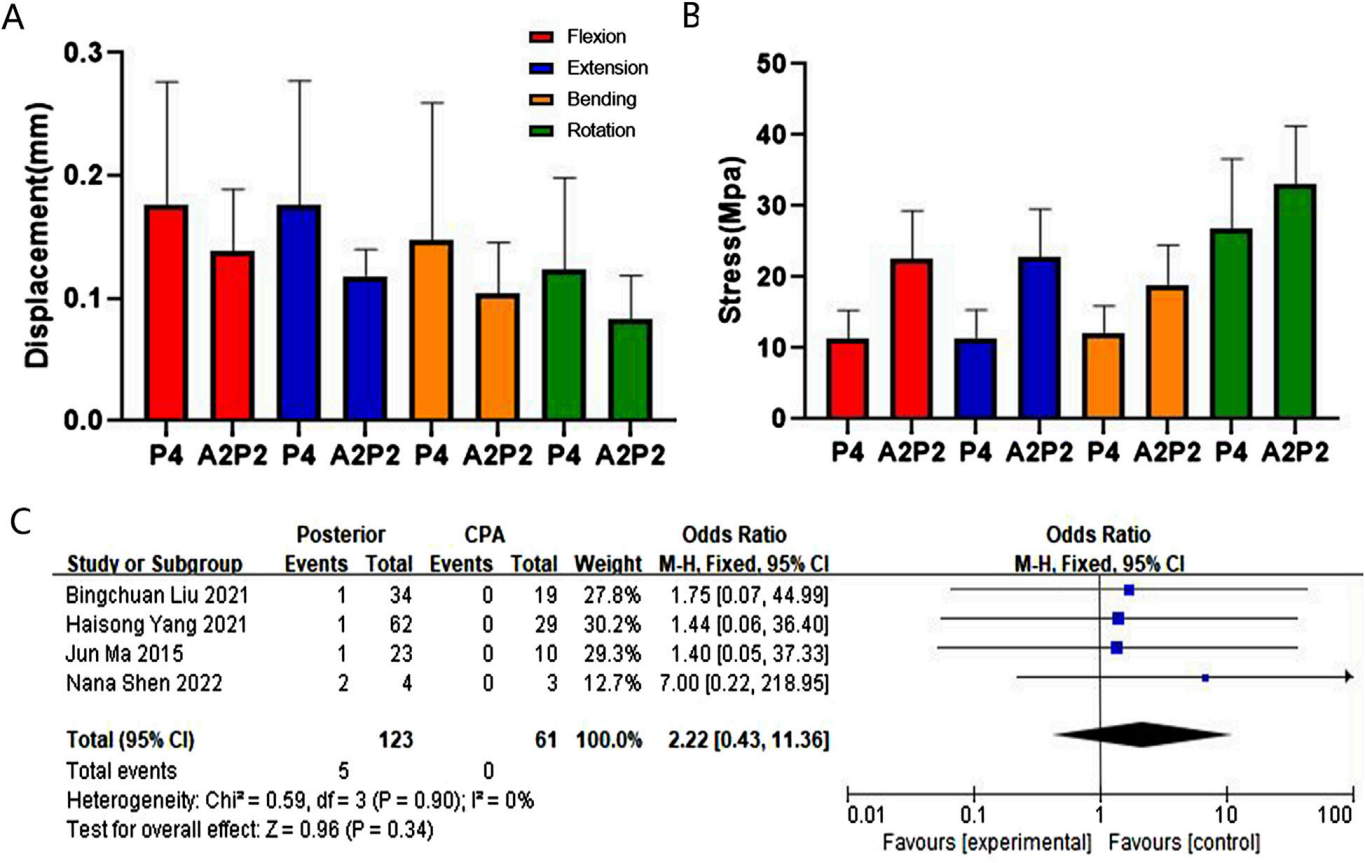
Comparison and verification of effects of posterior and CPA approaches. **(A)** Comparison of whole model displacement. **(B)** Comparison of screw stress values. **(C)** Meta-analysis of the screw loosening rate.

**FIGURE 7:**
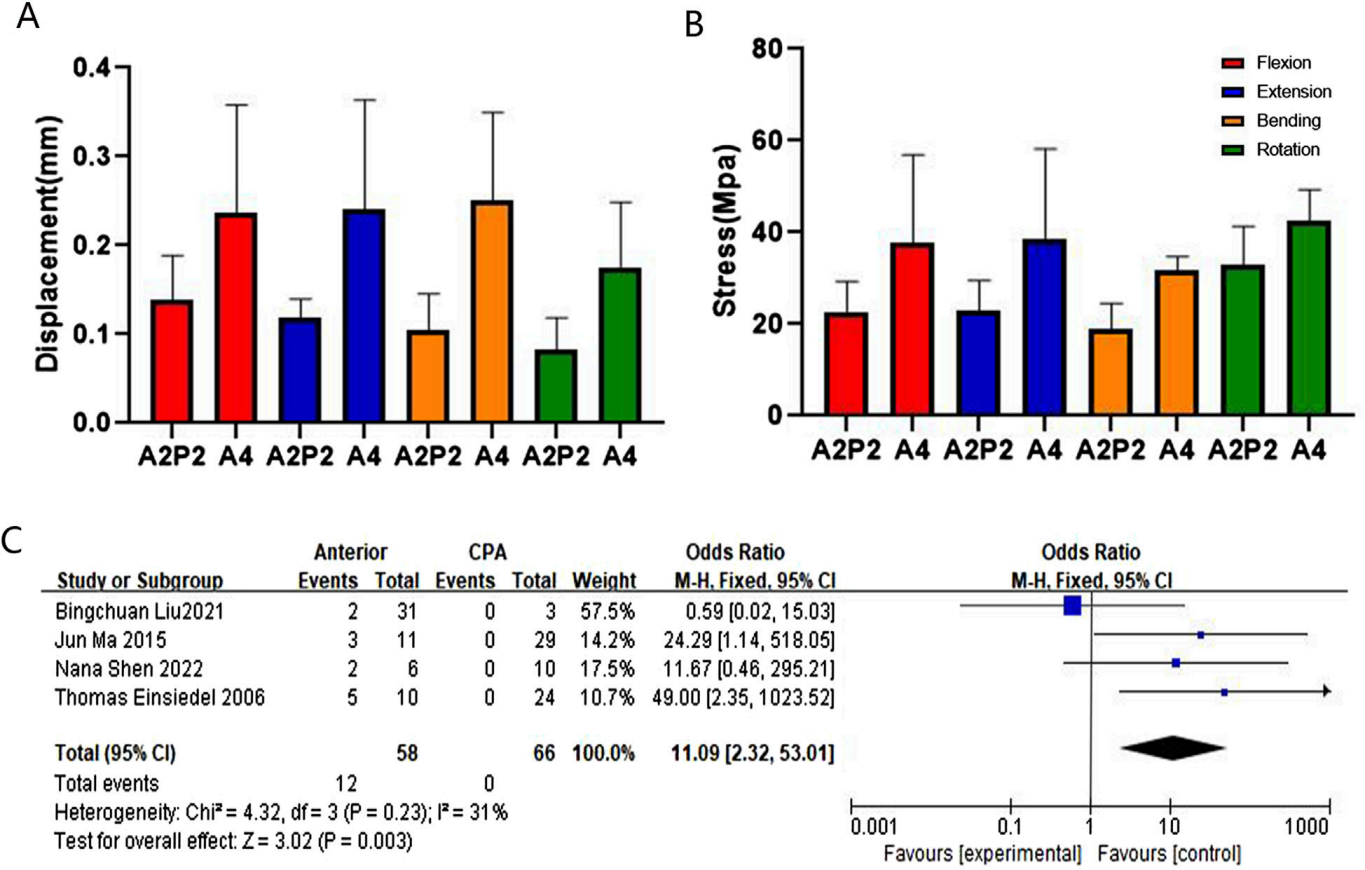
Comparison and verification of effects of anterior and CPA approaches. **(A)** Comparison of whole model displacement. **(B)** Comparison of screw stress values. **(C)** Meta-analysis of the screw loosening rate.

In terms of maximum displacement, during extending backward, A2 exhibited the highest displacement (0.67 mm), significantly higher than P2 at 0.25 mm, P2* at 0.22 mm, and P6 at 0.13 mm. The relationships between different fixation approaches for flexion, extension, bending, and rotation are in the following order: A2 > A4, P2 > P4 > P6, A2 > P2*, A4 > A2P2, P4 > A2P2, P2 > P2*. These finite element findings were further validated through meta-analysis (Figures 5, 6, 7, and Supplementary Figure S7).

## 4 Discussion

The current study measured and analyzed the bone distribution characteristics of the cervical vertebrae in patients with ASCF and accurately simulated these characteristics using an ASCF model based on HU values. This model more closely resembles the actual condition of the patients with ASCF in terms of shape and stress analysis. The finite element analysis results of different fixation approaches obtained from this model align with our meta-analysis findings.

The results revealed that AS cervical vertebrae exhibit more severe osteoporosis compared to the lateral masses, with a gradual decrease in bone density from top to bottom. The bone distribution in AS is distinct from that of normal cervical vertebrae, and traditional finite element methods with uniform material assignment cannot accurately simulate this feature. Utilizing CT data from ASCF patients, HU values were determined for each volume element of the model and gradient values assigned based on the HU value-density-elastic modulus conversion formula, which accurately simulated the characteristics of ASCF osteoporosis and ligament ossification.

The model based on HU values has some anatomical advantages over traditional models. The AS cervical spine undergoes a bamboo-like transformation due to ossification of the ALL, PLL, CL, and annulus fibrosus. The proposed model, featuring a multi-shell structure, mirrors this bamboo-like transformation, and its sagittal position significantly resembles the sagittal position on CT imaging. The range of motion of the HU-based ASCF model is consistent with that of traditional methods. It confirms that the new method could effectively simulate ligament ossification.

In ASCF, the associated osteoporosis and ligamentous calcification make the spine susceptible to fractures due to minor external forces. Spinal stability predominantly depends upon on a stable internal fixation. Postoperatively, even minor displacement can result in critical screw loosening, especially in patients with severe osteoporosis. The cumulative effect of these minor movements over time substantially increases this risk, and even millimeter-level differences in displacement between different fixation methods are important for long-term fixation integrity.

The finite element analysis of the findings reveals that anterior fixation is more susceptible to loosening in cases of ASCF compared to posterior fixation. First, based on the HU value measurements, the HU value of the same cervical spine segment is significantly lower than that of the lateral mass, providing strong evidence that posterior fixation offers greater stability compared to anterior fixation. Additionally, our meta-analysis results indicate that the anterior screw loosening rate is higher than the posterior screw loosening rate. Furthermore, ASCF often presents as a cervical kyphosis deformity (Zhang et al., 2023b) and, according to the principle of internal fixation for long bone fractures, anterior fixation is located on the traction side of the cervical spine, which makes it more susceptible to loosening (Li et al., 2023). The aforementioned results suggest that models based on HU values are more accurate in stress analysis.

Results of the finite element analysis show thatA2P2 is superior to P4 in terms of maximum displacement (MD) of the entire model, but A2P2 is superior to P4 in terms of MS of the screw. The meta-analysis revealed no statistical significant difference in the loosening rate of posterior fixation screws compared with CPA approaches, but the included literature did not specify the number of fixed segments (Luksanapruksa et al., 2019; Liu et al., 2023; Sapkas et al., 2009; Liu B. et al., 2021). Posterior approaches are challenging for addressing severe anterior column injury in the cervical spine (Duan et al., 2015). CAP approaches have been found to effectively address severe anterior column injury (Sethy et al., 2022), although they are associated with significant trauma and difficult postoperative recovery. The maximum stress value of the screws in the long segment posterior approach is smaller than that of the CPA approach. Therefore, long-segment posterior fixation is suggested to be the preferred choice for ASCF without severe anterior column injury.

The finite element results indicate that P2* has greater stability than P2. Posterior fixation with cervical pedicle screws is an ideal fixation method in orthopedics (Cechin et al., 2023). However, the lack of well-defined anatomical landmarks makes screw placement more challenging in surgery for ASCF (Beucler, 2023). With the integration of navigation robots into computer-assisted cervical surgery, posterior fixation with cervical pedicle screws is fast emerging as a promising and preferred approach for ASCF.

## 5 Limitations

The sample size of AS patients included in this study is relatively small, and further follow-up studies are needed for validation. Furthermore, the newly developed ASCF model lacks clinical validation due to the restricted cervical spine motion in patients after ASCF surgery. Finally, due to the limited availability of AS cadaver specimens, the scope of the current study remained limited to performing finite element analysis of ASCF.

## 6 Conclusion

Overall, it can be concluded that ASCF models based on HU values can better simulate bone distribution. Using a long posterior approach is the most stable approach for the treatment of ASCF. When the anterior column of the cervical spine is severely injured, it is recommended to employed CPA approaches rather than simple anterior approaches.

## Data Availability

The datasets presented in this study can be found in online repositories. The names of the repository/repositories and accession number(s) can be found in the article/Supplementary Material.

## Abbreviations

ASCF, ankylosing spondylitis cervical spine fracture; HU, Hounsfield unit; MS, maximum stress value; MD, maximum displacement; CPA, combined posterior and anterior; AS, ankylosing spondylitis; ALL, anterior longitudinal ligament; PLL, posterior longitudinal ligament; CL, capsule ligament; LF, ligamentum flavum; ISL, interspinous ligament; ROI, region of interest.
