# Supplementary material for: Evaluation of the effect of cervical spine bone distribution on fixation in ankylosing spondylitis
Source: Front Bioeng Biotechnol. 2025 Feb 27;13:1430047. doi: 10.3389/fbioe.2025.1430047 (PMC11903474; doi:10.3389/fbioe.2025.1430047)
Supplement: Supplementary file 1 [file Table1.docx]

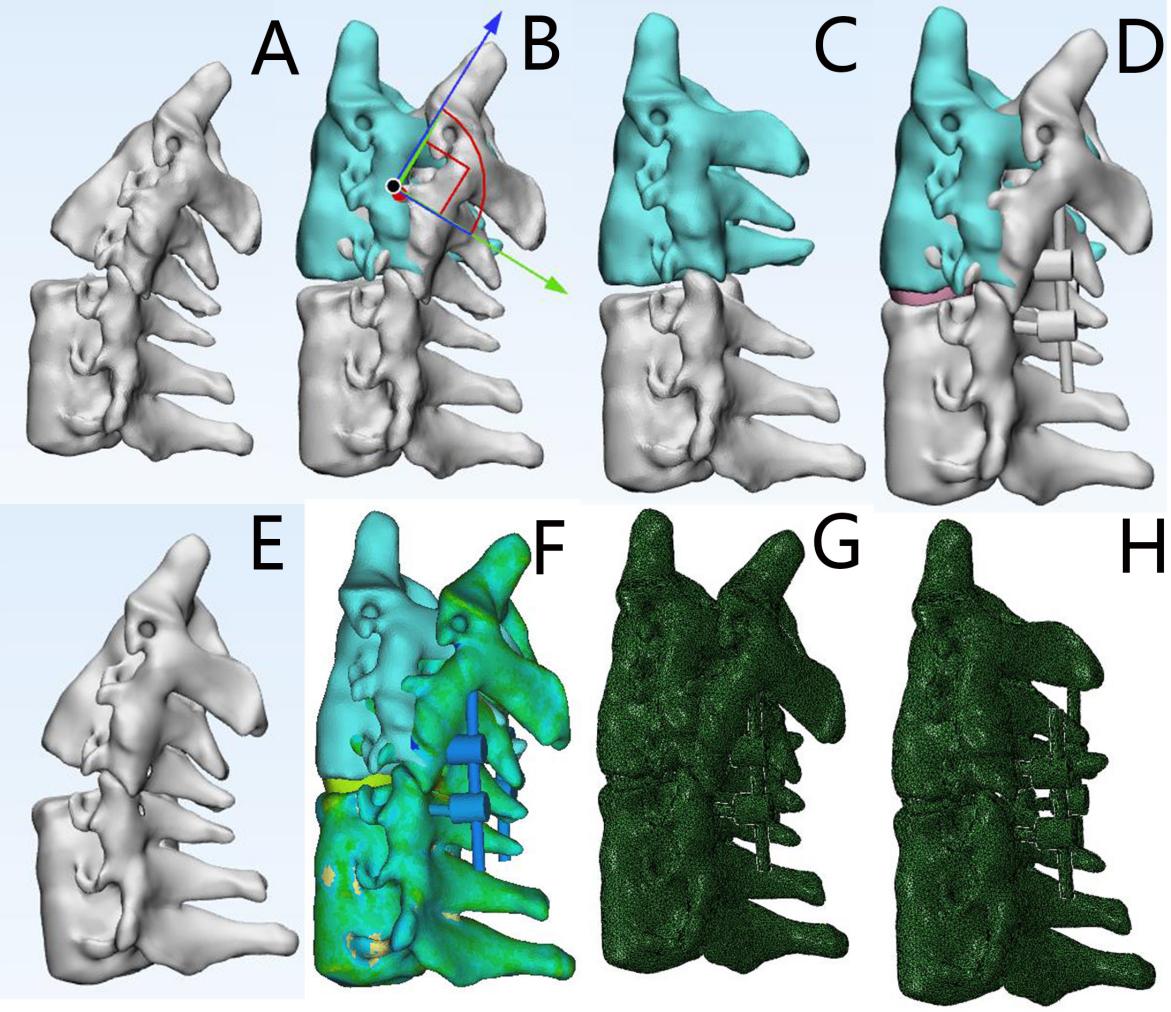


**Supplementary Figure1. ASCF model for fracture dislocation based on HU value assignment.** (A)ASCF model for fracture dislocation. (B)Fracture reduction with interactive translation and rotation. (C) Reset ASCF model. (D) Boolean operations on internal fixation and ASCF model. (E)Move the model back to its original position. (F)Dislocation model based on HU value assignment in Mimics. (G) The dislocation model overlaps with the reset model through geometric constraints in Abaqus. (H)ASCF model for fracture dislocation based on HU value assignment.

**
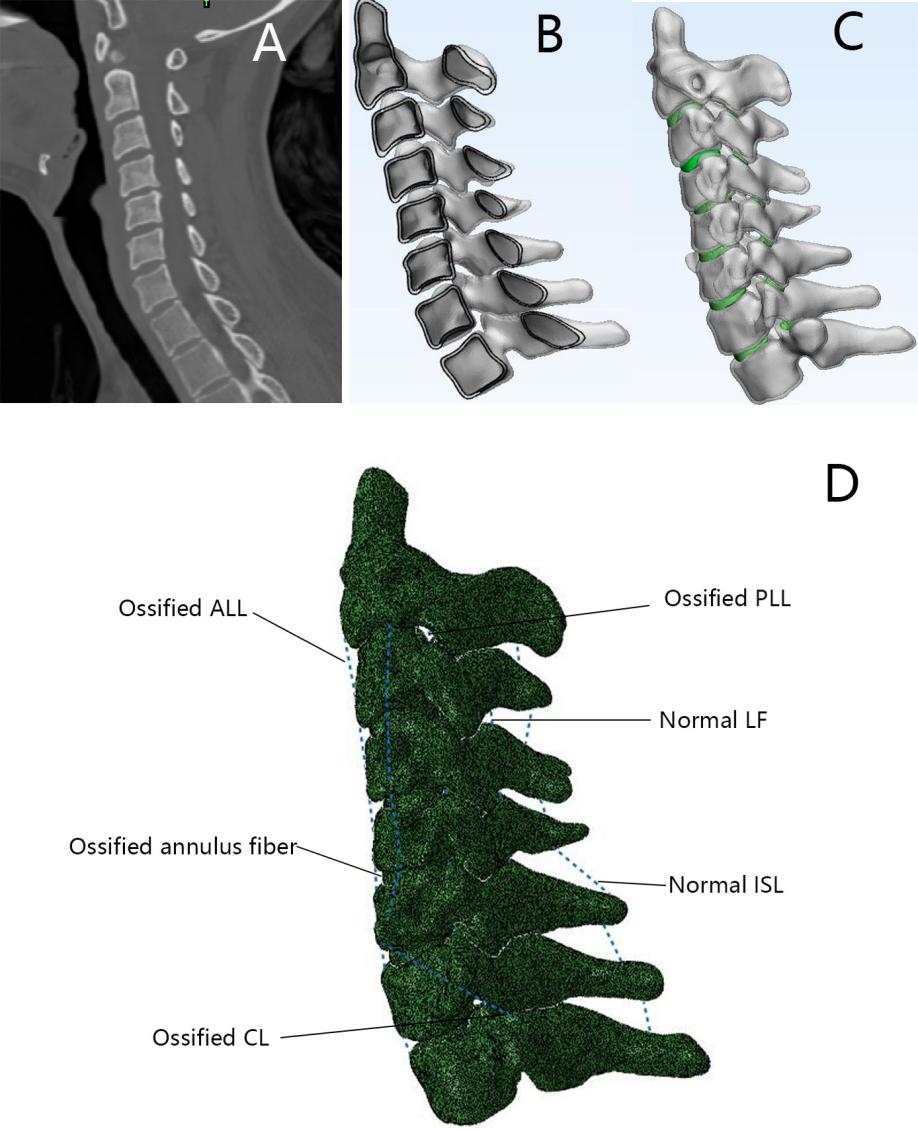
**

**Supplementary Figure2. ASCF model established by traditional method.** (A) Normal sagittal cervical imaging. (B) Establishment of cortical and cancellous bone structures. (C)Uniform assignment of models.(D)The model of ASCF ligament.

**
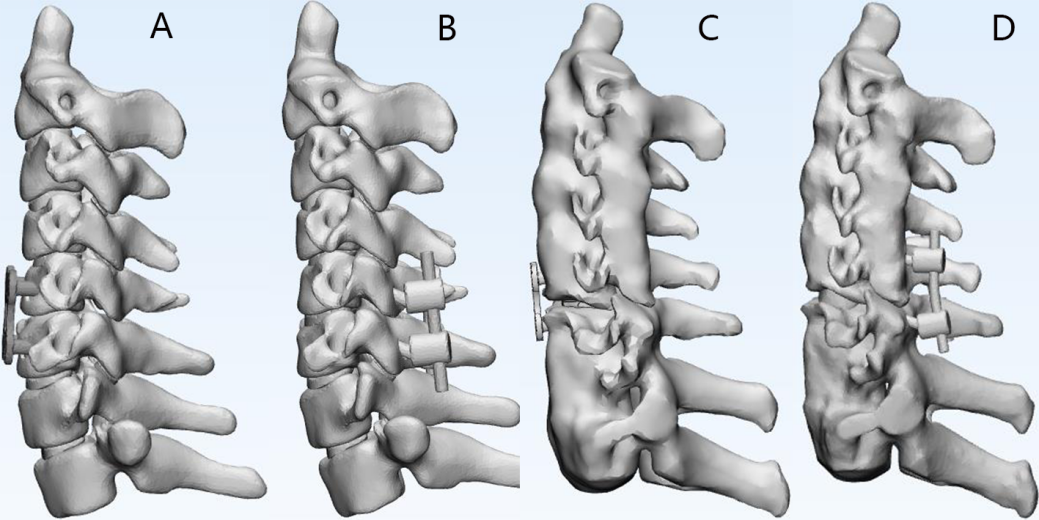
**

**Supplementary Figure 3** Comparison of screw stress values between traditional and HU value methods . (A) A2 constructed by traditional methods. (B) P2 constructed using traditional methods (C) A2 constructed using the HU value method. (D) P2 constructed using the HU value method.

**
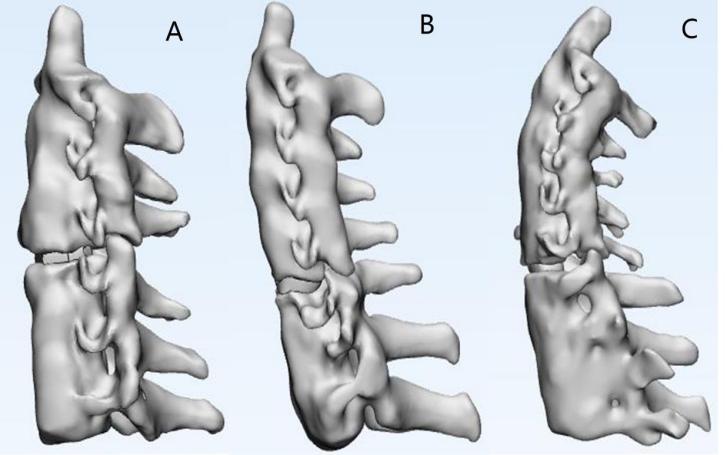
**

**Supplementary Figure 4*.***Multiple ASCF models based on HU values (A) C4-C5. (B) C5-C6. (C) C6-C7

**
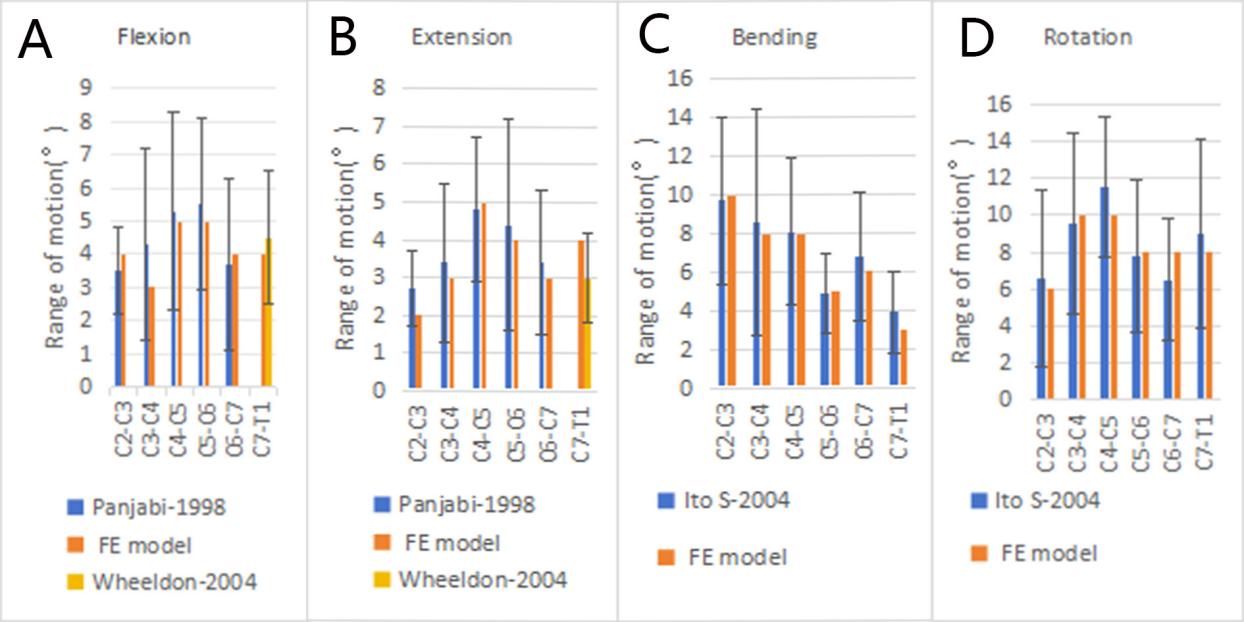
**

**Supplementary Figure5*.*** Validation of the validity of normal cervical C2-T1 established by traditional methods


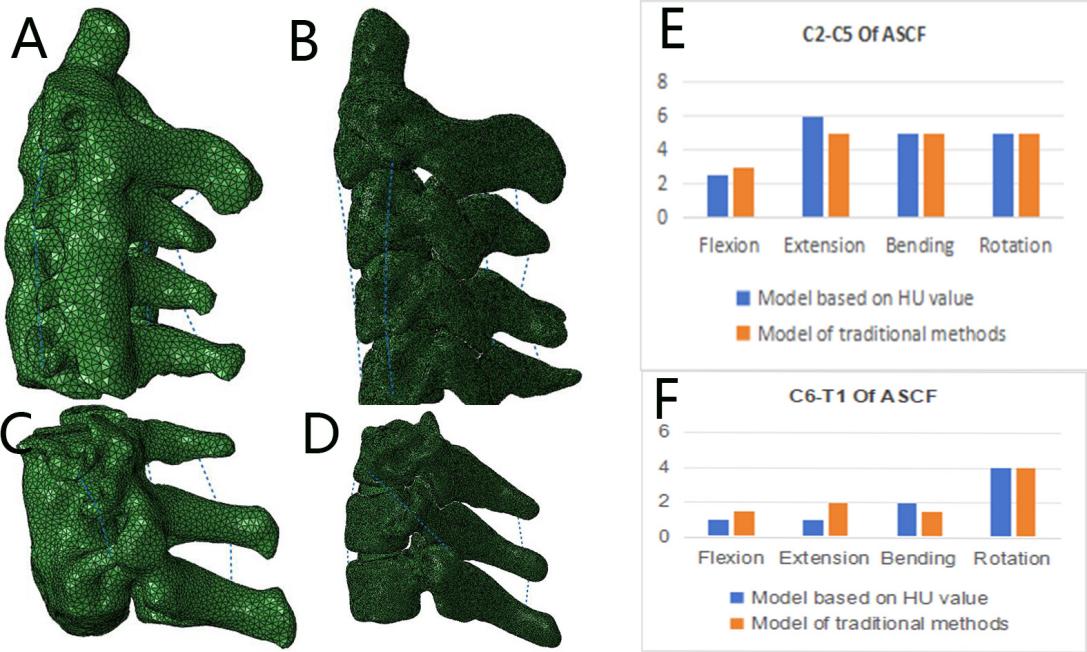


**Supplementary Figure 6*.* Comparison the range of motion of ASCF models constructed using different methods.** (A) C2-C5 segments of ASCF constructed based on HU value. (B) C2-C5 segments of ASCF constructed using traditional methods. (C) C6-T1 segments of ASCF constructed based on HU value. (D) C6-T1 segments of ASCF constructed using traditional methods. (E) The range of motion of C2-C5 of ASCF using different methods. (F) The range of motion of C6-T1 of ASCF using different methods.


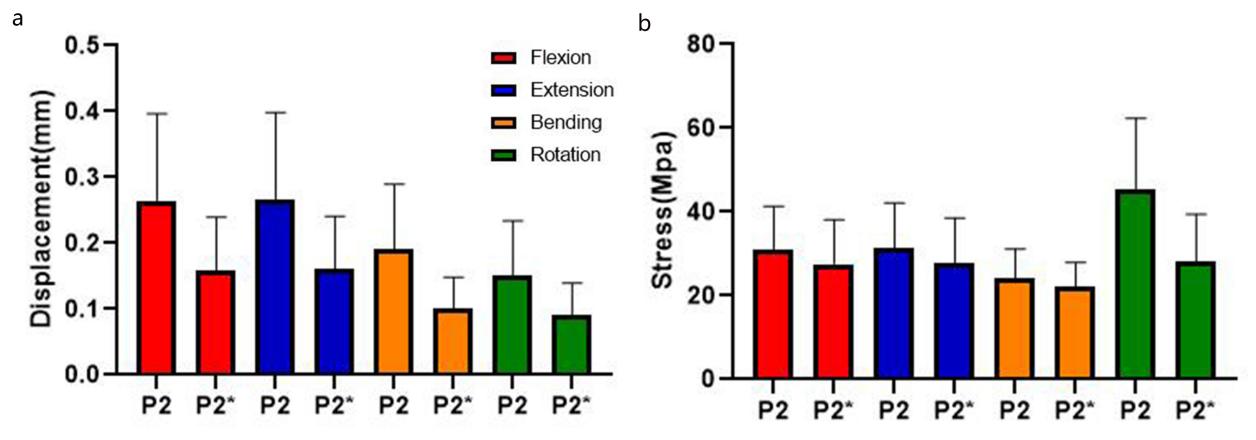


**Supplementary Figure7.Comparison of effects of P2 and P2***. (A) Comparison of whole Model Displacement. (B) Comparison of screw stress values.
