# Supplementary material for: Evaluation of the effect of cervical spine bone distribution on fixation in ankylosing spondylitis
Source: Front Bioeng Biotechnol. 2025 Feb 27;13:1430047. doi: 10.3389/fbioe.2025.1430047 (PMC11903474; doi:10.3389/fbioe.2025.1430047)
Supplement: Supplementary file 2 [file Table2.docx]

**HU value measurement data**


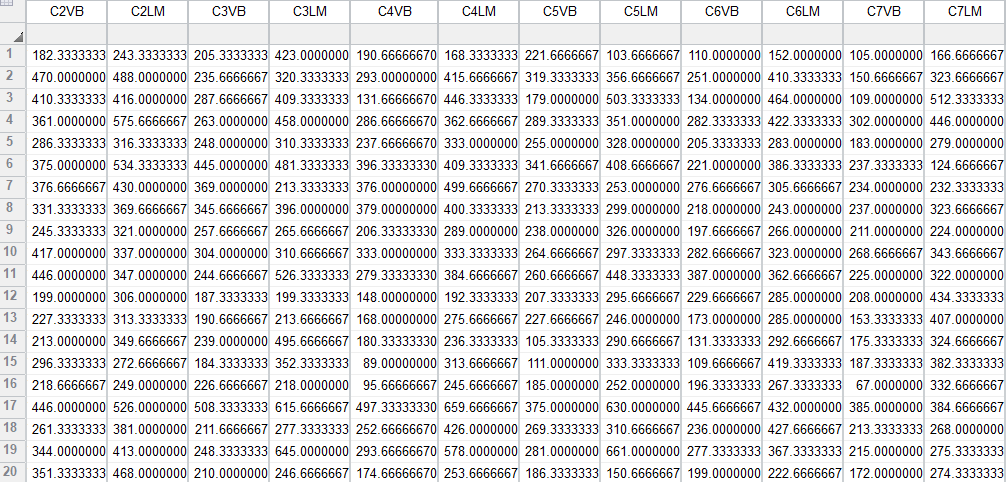


**The stress of screws ASCF model by tradition methods on 2Nm**

|  | Flension | Exention | Bending | Rotation |
| --- | --- | --- | --- | --- |
| A2 | 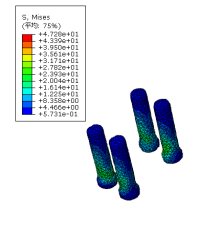 | 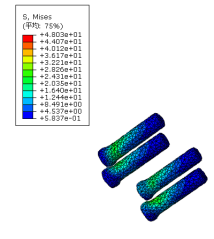 | 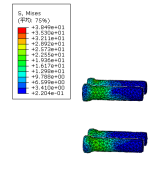 | 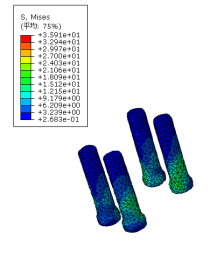 |
| P2 | 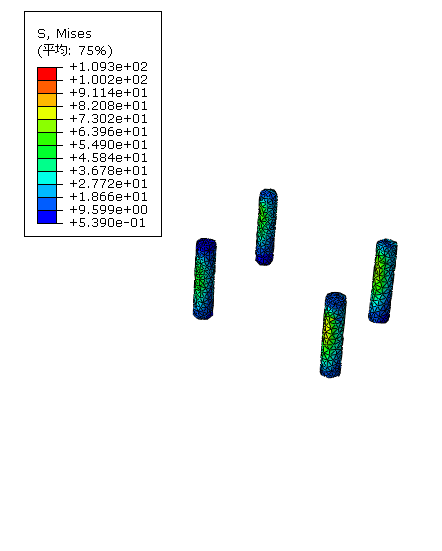 | 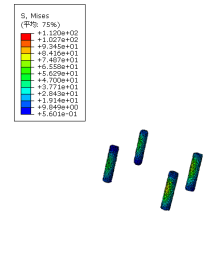 | 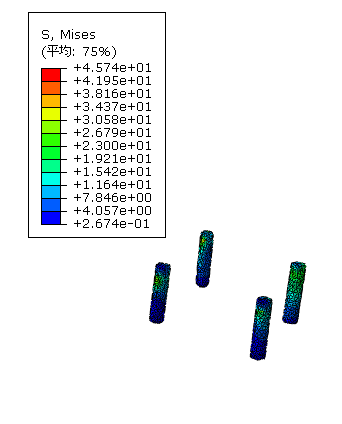 | 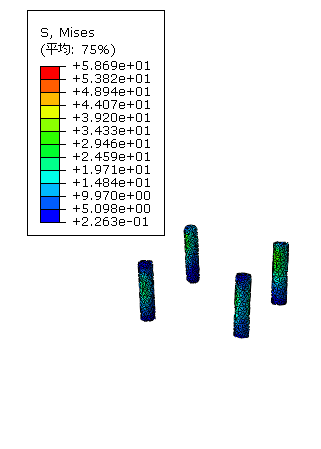 |

**1.The range of motion of C2-T1 model by tradition methods on 2Nm**

| Flension | Exention | Bending | Rotation |
| --- | --- | --- | --- |
| 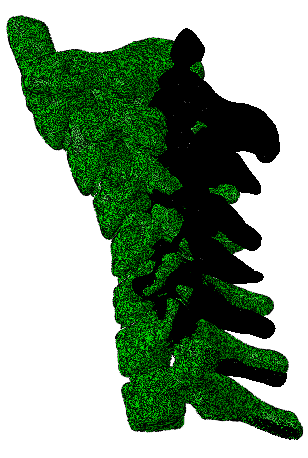 | 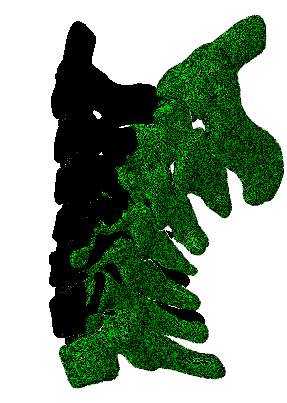 | 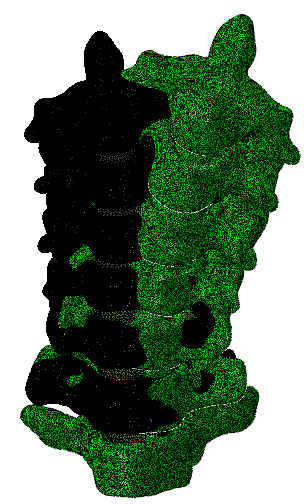 | 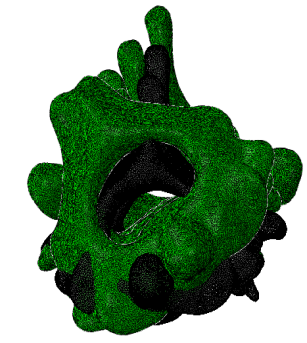 |

**2.The range of motion of C2-C5 ASCF model based on HU value on 100Nm**

| Flension | Exention | Bending | Rotation |
| --- | --- | --- | --- |
| 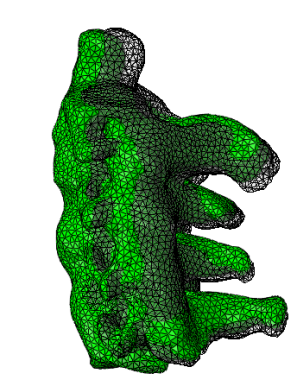 | 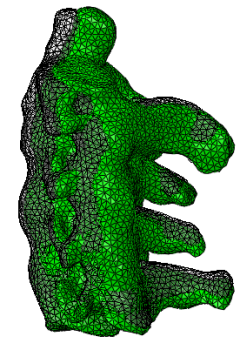 | 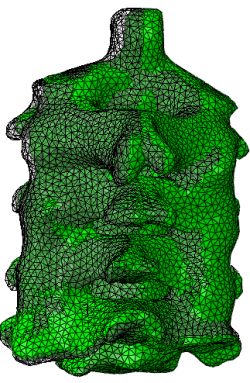 | 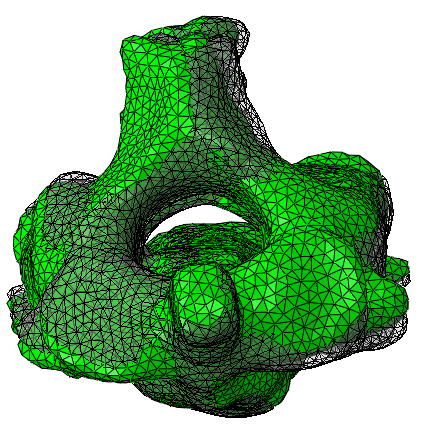 |

**3.The range of motion of C6-t1 ASCF model based on HU value on 100Nm**

| Flension | Exention | Bending | Rotation |
| --- | --- | --- | --- |
| 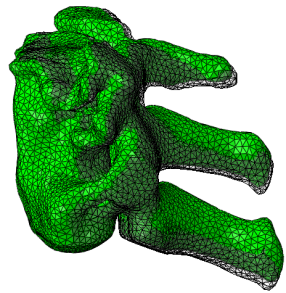 | 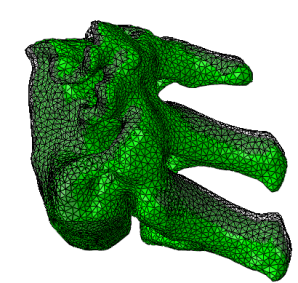 | 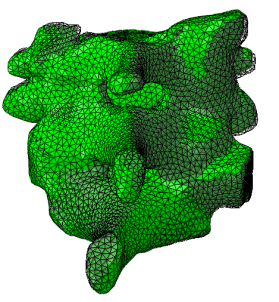 | 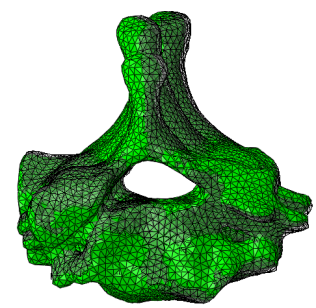 |

**4.The range of motion of C2-C5 ASCF model by tradition methods on 100Nm**

| Flension | Exention | Bending | Rotation |
| --- | --- | --- | --- |
| 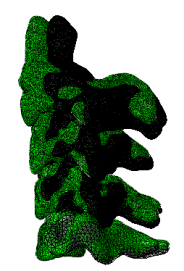 | 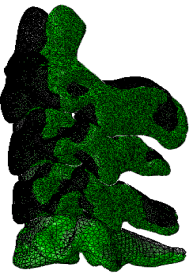 | 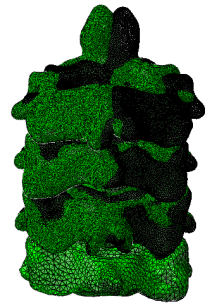 | 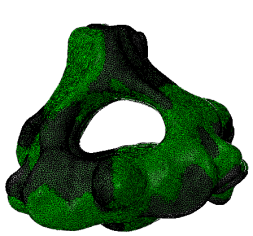 |

**5.The range of motion of C6-T1 ASCF model by tradition methods on 100Nm**

| Flension | Exention | Bending | Rotation |
| --- | --- | --- | --- |
| 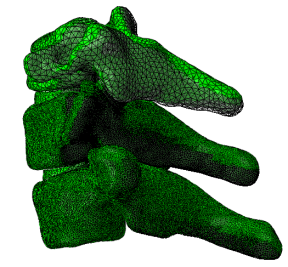 | 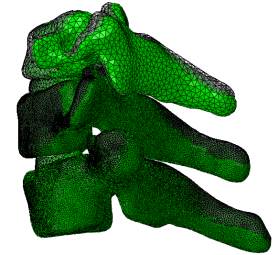 | 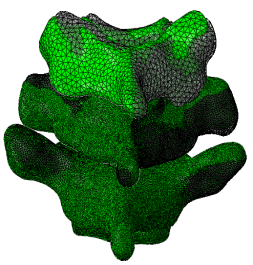 | 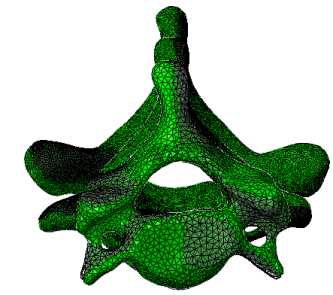 |

| **Model1 displacement** | Flexion | extension | bending | rotation |
| --- | --- | --- | --- | --- |
| A2 | 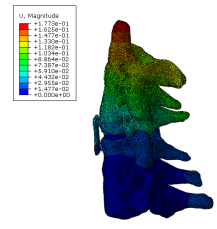 | 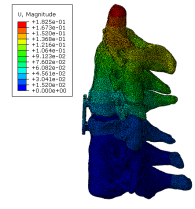 | 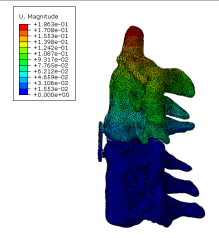 | 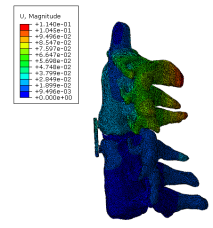 |
| A4 | 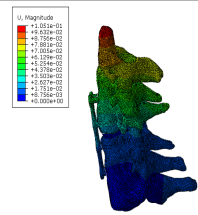 | 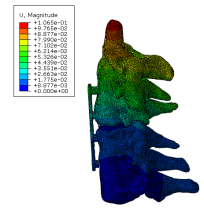 | 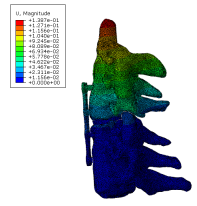 | 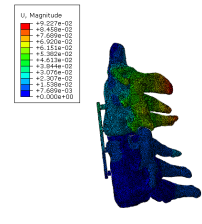 |
| P2 | 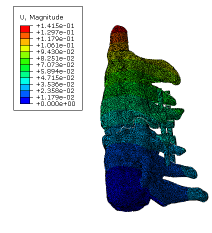 | 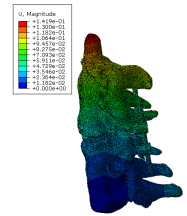 | 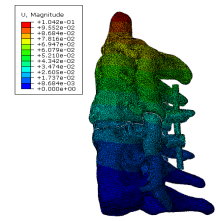 | 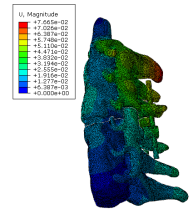 |
| P4 | 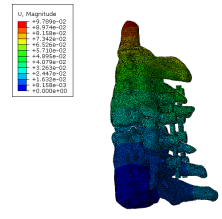 | 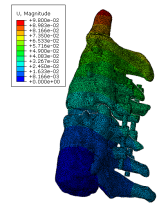 | 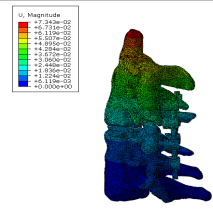 | 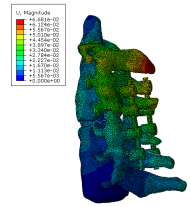 |
| P6 | 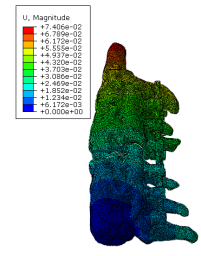 | 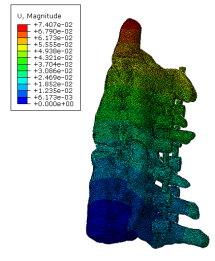 | 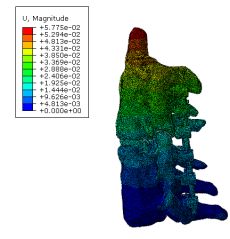 | 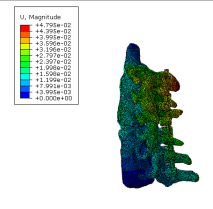 |
| P2’ | 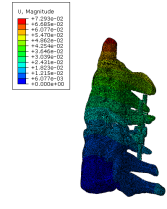 | 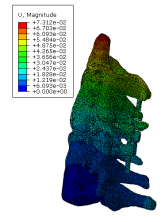 | 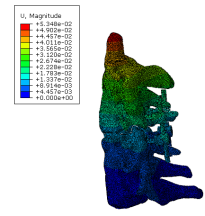 | 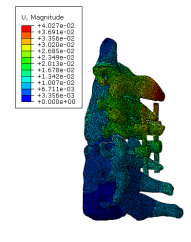 |
| A2P2 | 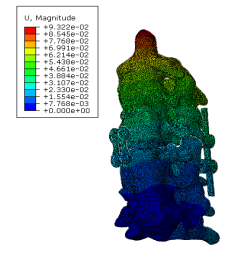 | 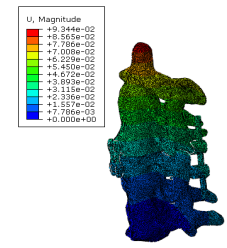 | 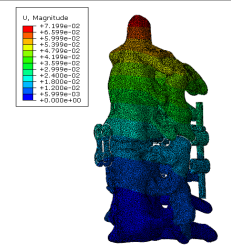 | 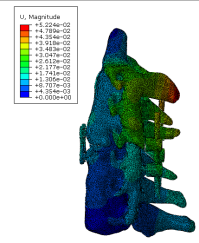 |
| **Model1**  **stress** | Flexion | extension | bending | rotation |
| A2 | 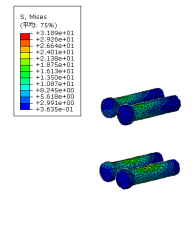 | 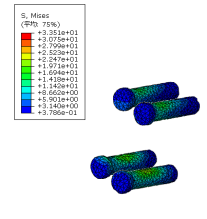 | 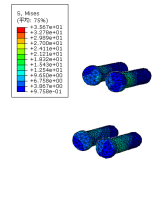 | 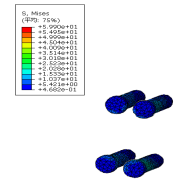 |
| A4 | 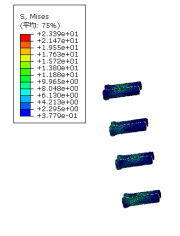 | 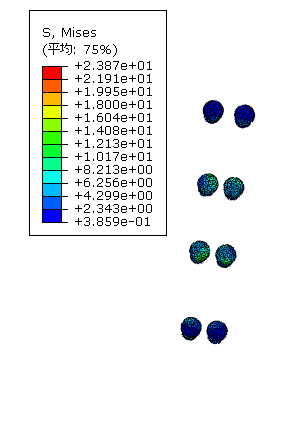 | 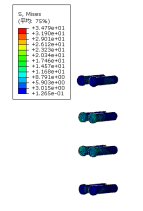 | 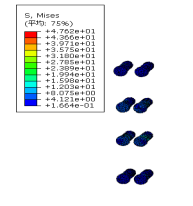 |
| P2 | 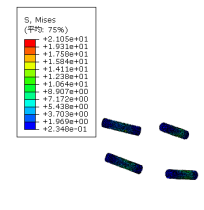 | 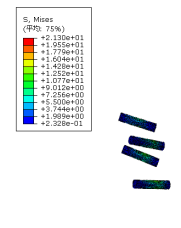 | 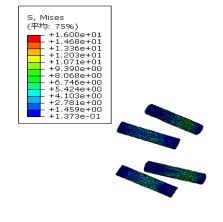 | 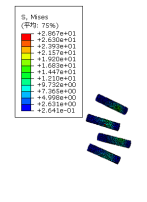 |
| P4 | 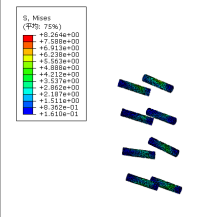 | 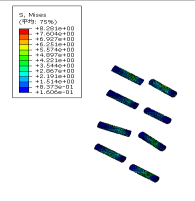 | 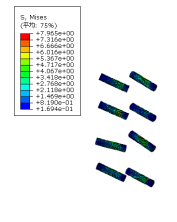 | 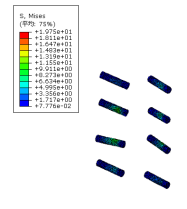 |
| P6 | 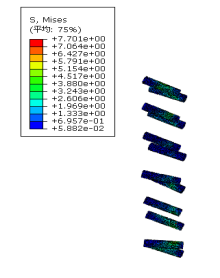 | 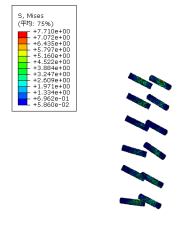 | 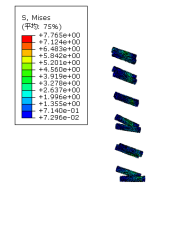 | 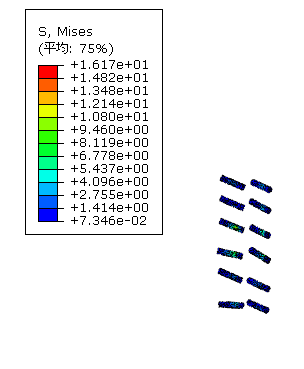 |
| P2’ | 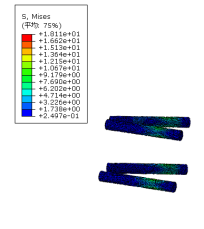 | 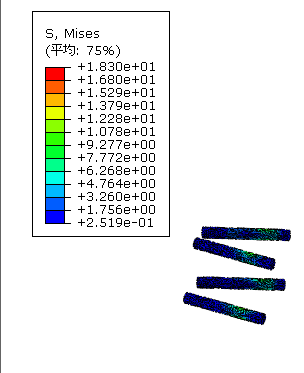 | 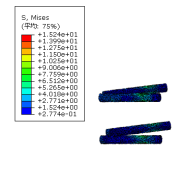 | 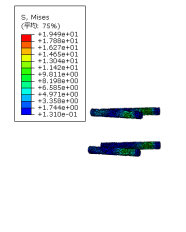 |
| A2P2 | 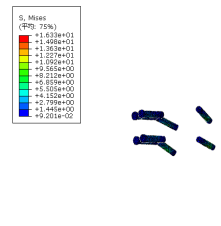 | 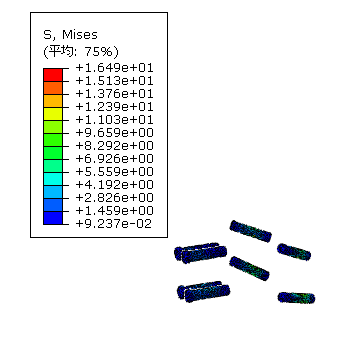 | 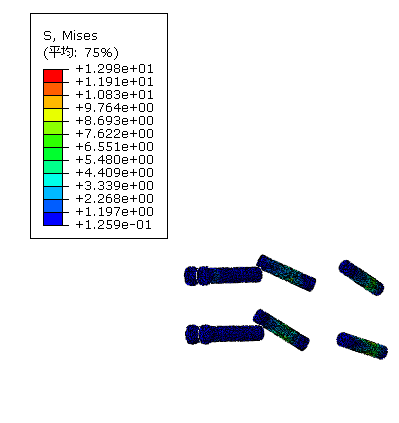 | 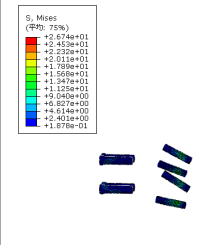 |

| **Model2**  **displacement** | Flexion | extension | bending | rotation |
| --- | --- | --- | --- | --- |
| A2 | 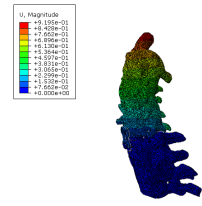 | 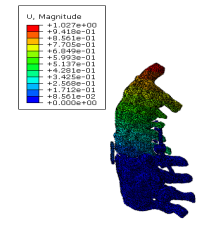 | 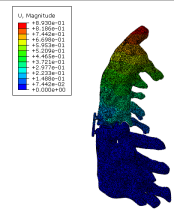 | 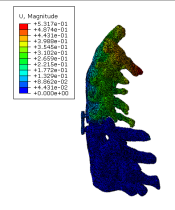 |
| A4 | 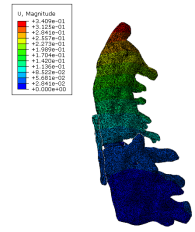 | 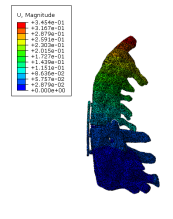 | 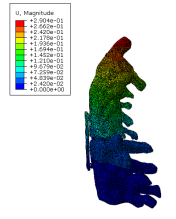 | 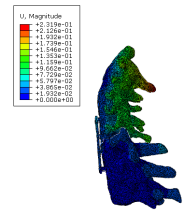 |
| P2 | 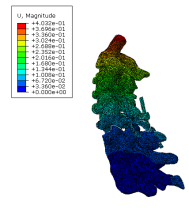 | 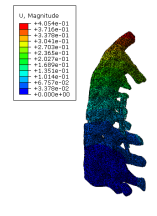 | 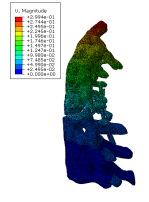 | 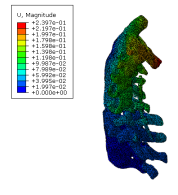 |
| P4 | 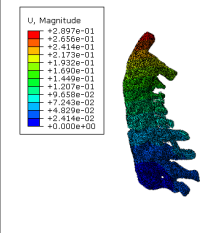 | 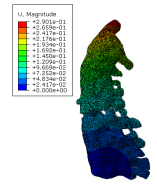 | 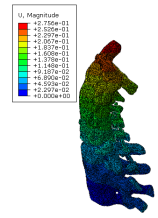 |  |
| P6 |  |  |  |  |
| P2’ |  |  |  |  |
| A2P2 |  |  |  |  |

| **Model2**  **stress** | Flexion | extension | bending | rotation |
| --- | --- | --- | --- | --- |
| A2 |  |  |  |  |
| A4 |  |  |  |  |
| P2 |  |  |  |  |
| P4 |  |  |  |  |
| P6 |  |  |  |  |
| P2’ |  |  |  |  |
| A2P2 |  |  |  |  |

| **Model3**  **displacement** | Flexion | extension | bending | rotation |
| --- | --- | --- | --- | --- |
| A2 |  |  |  |  |
| A4 |  |  |  |  |
| P2 |  |  |  |  |
| P4 |  |  |  |  |
| P6 |  |  |  |  |
| P2’ |  |  |  |  |
| A2P2 |  |  |  |  |

| **Model3**  **Stress** | Flexion | extension | bending | rotation |
| --- | --- | --- | --- | --- |
| A2 |  |  |  |  |
| A4 |  |  |  |  |
| P2 |  |  |  |  |
| P4 |  |  |  |  |
| P6 |  |  |  |  |
| P2’ |  |  |  |  |
| A2P2 |  |  |  |  |
